# In vitro photothermal therapy of pancreatic cancer mediated by immunoglobulin G-functionalized silver nanoparticles

**DOI:** 10.1038/s41598-024-63142-4

**Published:** 2024-06-22

**Authors:** Andreea Nedelcu, Teodora Mocan, Lavinia Ioana Sabau, Cristian Tudor Matea, Flaviu Tabaran, Teodora Pop, Cristian Delcea, Ofelia Mosteanu, Lucian Mocan

**Affiliations:** 1 3rd Surgery Clinic, “Iuliu Hatieganu” University of Medicine and Pharmacy, ClujNapoca, Romania; 2 Nanomedicine Department, Regional Institute of Gastroenterology and Hepatology, ClujNapoca, Romania; 3 Physiology Department, “Iuliu Hatieganu” University of Medicine and Pharmacy, ClujNapoca, Romania; 4 Department of Biosciences, University of Salzburg, Hellbrunnerstraße 34, 5020 Salzburg, Austria; 5 Department of Pathology, Sciences and Veterinary Medicine, University of Agricultural, ClujNapoca, Romania; 6 Department of Gastroenterology, “Iuliu Hatieganu” University of Medicine and Pharmacy, ClujNapoca, Romania; 7 Department of Forensic Medicine, “Iuliu Hatieganu” University of Medicine and Pharmacy, ClujNapoca, Romania

**Keywords:** Pancreatic cancer, Silver nanoparticles, Immunoglobulin G, Treatment, Pharmaceutics, Drug development, Biotechnology, Nanobiotechnology, Nanoparticles

## Abstract

Pancreatic cancer is one of the most aggressive forms of cancer, and treatment options are limited. One therapeutic approach is to use nanoparticles to deliver the active agent directly to pancreatic cancer cells. Nanoparticles can be designed to specifically target cancer cells, minimizing damage to healthy tissues. Silver nanoparticles have the unique ability to absorb light, especially in the near-infrared (NIR) region. In this study, silver nanoparticles functionalized with IgG molecules were synthesized and administered to pancreatic cancer cell lines. Subsequently, the cells were photo-excited using a 2 W 808 nm laser and further examined in PANC-1 pancreatic cancer cell lines. Flow cytometry and confocal microscopy combined with immunochemical staining were used to examine the interaction between photo-excited silver nanoparticles and pancreatic cancer cells. The photothermal therapy based on IgG-functionalized silver nanoparticles in pancreatic cancer induces dysfunction in the Golgi apparatus, leading to the activation of the caspase-3 apoptotic pathway and ultimately resulting in cellular apoptosis. These findings suggest that our proposed IgG nanoparticle laser treatment could emerge as a novel approach for the therapy of pancreatic cancer.

## Introduction

Pancreatic cancer is a highly deadly solid tumor in humans. Moreover, most patients with pancreatic cancer are resistant to all existing cytostatic treatments. There is a dire need for the development of new therapeutic strategies to overcome these challenges^1^.

Curative treatment options for this aggressive cancer are limited^2^. Surgical removal of the tumor is the only option for a cure, but it is feasible in only a small percentage of patients^3^. In this context, scientists are investigating the potential use of nanoparticles for treating pancreatic cancer^4^.

In recent years, researchers have been exploring the use of nanoparticles and lasers to enhance hyperthermia as a cancer treatment. This approach is known as laser-induced hyperthermia therapy. The idea is to use nanoparticles to deliver heat to cancer cells in a targeted and controlled manner.

One approach is to use nanoparticles to deliver chemotherapy drugs specifically to pancreatic cancer cells^5^. This technique has the potential to decrease the adverse effects associated with traditional chemotherapy by selectively targeting cancer cells and avoiding healthy cells. For example, researchers have developed nanoparticles that specifically focus on cancer cells that have an overexpression of certain receptors, such as albumin receptors^6^.

Another approach is to use nanoparticles for imaging and diagnosis of pancreatic cancer. Nanoparticles can be de- signed to accumulate in tumor tissues, allowing for earlier and more accurate detection of pancreatic cancer. Additionally, nanoparticles can be used to track the distribution of drugs in the body, providing insights into drug delivery and helping to optimize treatment strategies^7^.

Finally, nanoparticles can also be used for hyperthermia therapy, as discussed earlier. By delivering nanoparticles to the tumor site and then heating them with a laser, cancer cells can be selectively destroyed, potentially improving treatment outcomes for pancreatic cancer patients^8^.

While nanoparticles hold great promise for pancreatic cancer treatment, more research is needed to optimize their effectiveness and ensure their safety for use in humans^9^. Nonetheless, this area of research is a rapidly growing field with potential to revolutionize pancreatic cancer treatment.

Cancer treatment mediated by nanoparticles holds great hope for the development of future therapies. Plasmonic photothermal therapy converts photon energy into heat at micro-scale level using nanoparticles with this special property^10^. Of these nanoparticles, silver nanoparticles exhibit strong optical absorbance, have low toxicity and can be easily bio-functionalized with active pharmaceutics agents or carriers^11^.

The fundamental objective of cancer-therapy nanomedicine is to attain tissue specificity, leading to the exclusive accumulation of nanoparticles within the malignant tissue. Considerable research endeavors have been dedicated to the creation of targeted cancer therapies involving the conjugation of antibodies. More specifically, highly proliferative tumor cells display an overabundance of particular receptors on their cell membrane, which can be utilized as selective targets for the antibody-conjugated nanoparticles. Recent studies have presented compelling evidence of a strong association between heightened levels of Immunoglobulin G antibodies and the promotion of cancer cell proliferation, invasion, and unfavorable clinical outcomes in cancer patients. Immunoglobulin G molecules consist of two light chains and two heavy chains arranged in a Y-shaped structure. The variable regions of the arms of Immunoglobulin G identify specific antigens through the V(D)J recombination process, a crucial mechanism in therapeutic interventions like anti-cancer treatment. Gene segments undergo reorganization and fusion to generate the constant region, which determines the class of Ig. The presence of Immunoglobulin G has been detected in tumor tissues across various organs, including pancreatic cancer. A compelling research study demonstrated that obstructing tumor-cell-derived Immunoglobulin G impeded the progression of tumor cells^12^.

**Figure 1:**
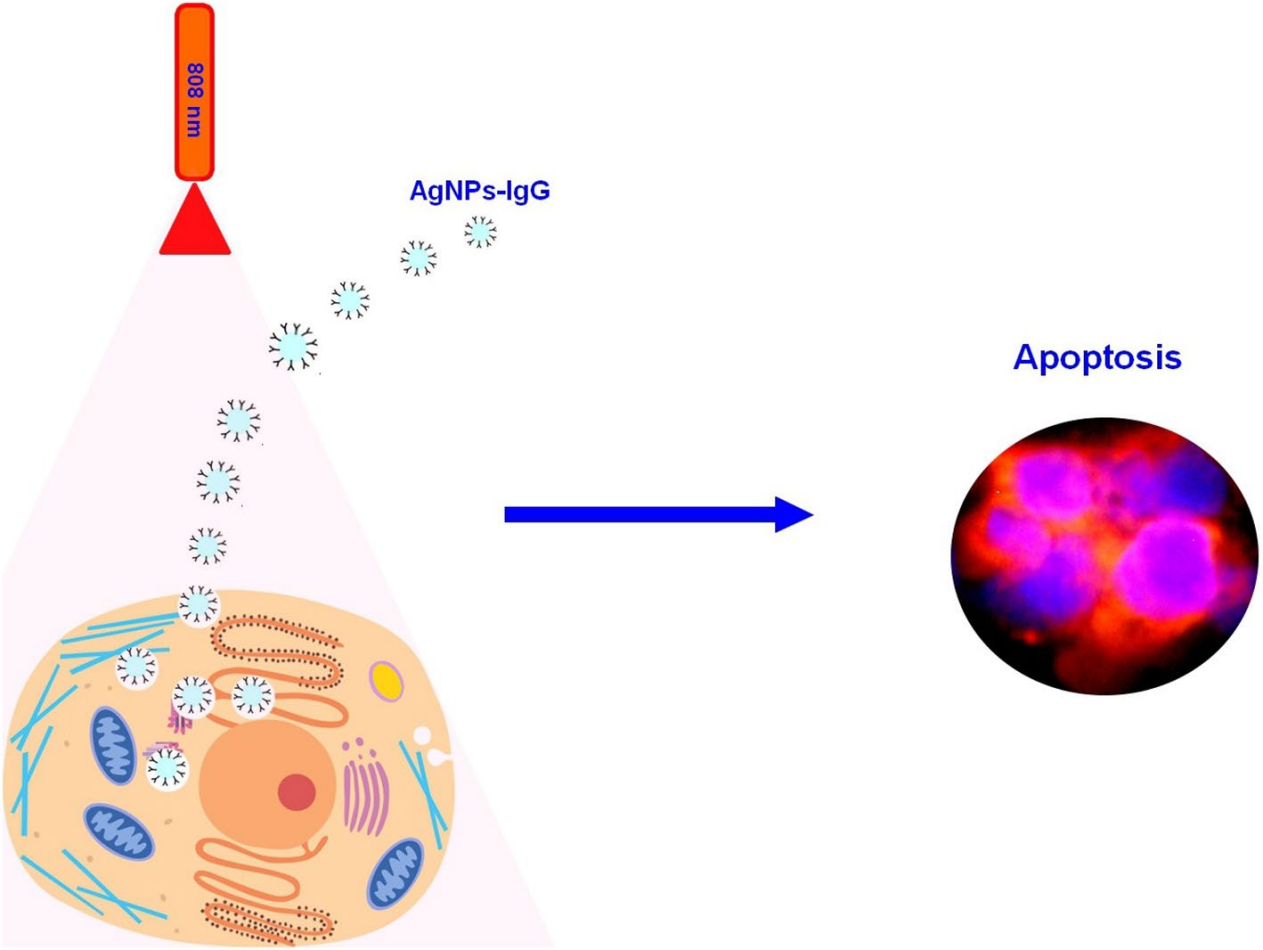
The schematic illustration of the proposed treatment.

Driven by these scientific data and considering that silver nanoparticles have with strong resonances in NIR to operate as photo-thermal agents^13,14^, we have developed and tested the photo-thermal therapeutic potential of an AgNPs-IgG nano- bio system against a very aggressive tumor such as pancreatic cancer (Fig. 1).

## Methods

### Nanoparticle synthesis

Silver nitrate (AgNO_3_ 99.9%), sodium citrate (≥ 99%), *N*-(3-Dimethylaminopropyl) -*N*^*j*^-ethylcarbodiimide (EDC) and *N*-Hydroxysuccinide.

Immunoglobulin G (IgG) was purchased from Kedrion (Lucca, Italy) and further purified using Pierce concentrators with a 30 kDa molecular cut-off Calcium folinate (CF) was obtained from Actavis (Romania). All used glassware was washed with aqua regia (HCl: HNO_3_, 3: 1 v/v) before use.

The nanoparticle synthesis and functionalization was done according to the protocol previously described by us with minor modifications^15^. Briefly, following the reduction of silver ions in the presence of sodium citrate, silver nanoparticles (AgNP) were synthesized as follows: 20 mg AgNO_3_ were dissolved in 100 mL H_2_O dist. and the solution was brought to boiling.. Next, 5 mL sodium citrate solution (0.5%) was added rapidly under continuous stirring, and the reaction was allowed to continue until a pale yellow color was obtained.

Functionalization of silver nanoparticles with Immunoglobulin G was performed using calcium folinate (CF) following the reaction of 6 mL Immunoglobulin G solution (5 mg/mL) with 3 mL sol. NHS (30 mg/mL) and 3 mL sol. EDC (30 mg/mL) under continuous stirring for 25 min at room temperature. Next, 2 mL of CF (3 mg/mL) was added and the reaction was allowed to continue for another 30 min. The purification of IgG-CF moieties was performed using centrifugal concentrators with a molecular cut- off of 30 kDa at 4000RPM/60 min. The IgG-CF conjugate was redissolved in 12 mL of distilled H_2_O and mixed with AgNPs while stirring continuously for 60 min. Finally, the AgNPs biosystem was separated by centrifugation at 13200RPM/20 min. and re-dispersed in H2O at various concentrations.

To define the functional groups and adsorbed molecules at the surface AgNPs nano-bio system and surface chemistry morphology, UV–Vis spectroscopy investigations were performed with a Shimadzu UV-1800 spectrophotometer. The 800–200 nm range was used for spectra recordings, with a spectral resolution of 0.5 nm. OriginPro 2020 7.0 software was used to normalize the spectra.

To accurately measure the size distribution profiles of the obtained silver nanoparticles particles, dynamic light scat- tering (DLS) measurements were performed using a Zetasizer—Nano S90 instrument (Malvern Instruments, Westborough, UK) at 20 °C and a diffraction angle of 90° and a refractive index of 0.20. The material absorption index used was 3980.

To perform the in-situ analysis of interfaces and to investigate the surface adsorption of IgG groups on silver nanoparti- cles, attenuated total reflectance Fourier transform IR spectroscopy (ATR-FT-IR) spectroscopy investigations were performed using a Perkin-Elmer Spectrum Two instrument equipped with an ATR stage. All recorded spectra were processed with Spectrum 10 software.

To obtain qualitative and quantitative data on AgNPs physical properties including size, morphology and surface texture atomic force microscopy measurements were performed with a TT-AFM Workshop instrument (AFM Workshop, CA, USA), equipped with non-contact ACTA-SS cantilevers (AppNano, CA, USA). The samples were deposited on a mica substrate using of a KLM SCC spin coater. The recorded data were processed using Gwyddion 2.36 software.

### Cells culture

The cells used in this study (Panc-1 and epithelial cells) were purchased from the European Cellular Culture Collection (ECCC) and further cultured in MEM culture medium (Minimum Essential Medium) with 10% fetal bovine serum at 37 °C in a humidified atmosphere of 5% CO_2_ using 25 cm^3^ plastic tissue culture vials (Corning) until reaching confluence.

### Microscopic analysis of cells

Following separation and trypsinization, the cells were placed on Lab-Tek Chamber Slide slides for real-time mi- croscopy analysis. We used for immunofluorescence microscopy a microscopic FSX- 100 Olympus microscopy while hyper- spectral analysis was performed using Cytoviva hyperspectral microscope.

### Laser treatment

The functionalized silver nanoparticles were added to cells at various concentrations (1, 5, 10 and 50 µg/mL) and further irradiated for 2 min using an 808 nm laser with a power of 2 W/cm^2^. The laser diode was placed in a vertical position above the plate and multiple irradiation spots were addressed in order to obtain a uniformity of the irradiation onto the surface of the cells-4-well culture plates (the cells were further analyzed by flow microscopy or cytometry).

### Annexin-V Cy3 Apoptosis Detection Assay (BioVision, Inc)

The process of cellular apoptosis suggested by phosphatidylserine translocation to the outer layer of the cell membrane was assessed using the Annexin-V Cy3 Apoptosis Detection Assay (BioVision, Inc.) according to the manufacturer’s protocol. Following treatment, the cells were cultured on 4 rooms glass slides (Milipore), incubated with Annexin V-Cy3 (5 min, dark) and then analyzed by microscopy using the rhodamine filter. Flow cytometry analyses were also performed. Annexin V was used for quantifying the cellular apoptosis with a Millipore flowcytometer. The counting level was below 1000 s^−1^ events and the events were collected at low speed, logarithmically (approx. 15 µl min^−1^).

### Golgi apparatus (AG), caspase 3 and Nucleus detection test (N)

To assess, the effects of our treatment on cellular morphology and physiology, multiple staining of cellular organelles was performed using the Organelle-ID RGB III Assay kit and the Caspase-3 fluorometric detection kit (Enzo Life Sciences). Briefly, 100 µL of test solution was administered to the cells. Next, the cells were treated with multiple staining solutions (100 µL/well, 30 min, 4 °C, in the dark). A second washing step was applied (3 × ice-cold medium), followed by immersion of the cells in a fresh ice-cold medium (37 °C, 30 min). Following washing with 100 µL wash buffer fixation (10% formalin, 5 min) the cells were detached and mounted on a coating lamella (water-based, Magnacol =). The fluorescence microscopy analysis was performed using standard filters to detect distinct fluorescent emission signals: FITC (Golgi apparatus), Rhodamine (caspase 3) and DAPI (core). (Mountant, Magnacol).

### Cell viability analysis after AgNPs-IgG treatment

The cellular viability following the treatment was assessed using MTT assay. Following the treatment, cells were incubated for 4 h with 0.8 mg/ml of MTT reagent diluted in serum free medium (DMEM). 1 ml DMSO solution was added under complete shaking for 10 min until total dissolution was achieved. Aliquots (150 µL) of the resulting solutions were transferred in 96-well plates and absorbance was obtained at 560 nm using the Victor 3 microplate reader.

### Statistical analysis

The obtained statistical data were expressed as mean (range). Because of the non-normality characteristics of the data, the Kolmogorov–Smirnov test was chosen. Wilcoxon test was used for the Comparison of data for the same concentration between two groups. An alpha error level < 0.05 was chosen for all tests. Data analysis was performed using SPSS statistical packages (Chicago, IL, USA), as well as the Excel application of the Microsoft Office platform.

## Results

### Synthesis and characterization of AgNPs-IgG nano-bio system,

**Figure 2:**
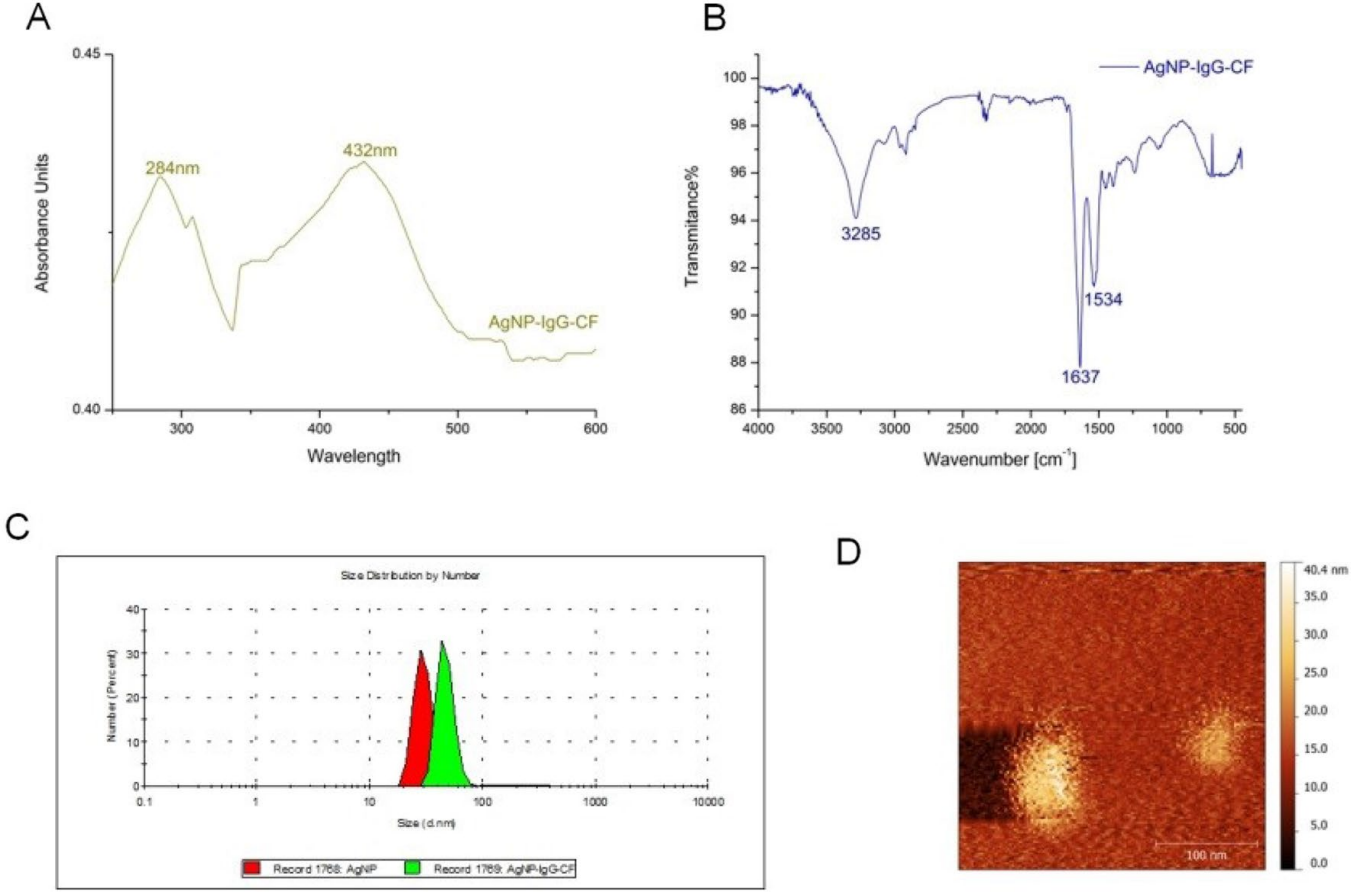
(**A**). Ultraviolet absorption spectrum of AgNPs-IgG complex. (**B**). IR spectra for CF, IgG, AgNP-IgG-CF, AgNP, IgG-CF. (**C**). Particle size distribution histogram of the IgG-CF functionalized AgNPs by DLS. AgNPs (red) and AgNP-IgG-CF (green). (**D**). AFM image of AgNP-IgG-CF bionanostructure. Abbreviations: AgNP, silver nanoparticle; IR, infrared; UV–Vis, ultraviolet visible; DLS, dynamic light scattering; AFM, atomic force microscopy.

Figure 2A displays the UV–Vis absorption spectra of the AgNP-IgG-CF solution. The absorbance peak observed at 432 nm is attributed to the bandwidth resonance phenomenon of silver nanoparticles, while the peak at 284 nm is ascribed to Immunoglobulin G molecules coupled to the AgNP surface during the functionalization stage.

In Fig. 2B, IR spectroscopy investigations were conducted to identify the secondary structure of proteins in the AgNPs bionanosystem. The absorbance bands were compared with standard values to identify functional groups^16^. The IR absorbance band at 1637 cm^−1^ is strongly associated with amide I, attributed to the ‘stretching’ vibrations of the C=O bond, and the band at 1534 cm^−1^ corresponds to amide II (vibrations of ’stretching’ of C–N bonds and ’bending of N–H bonds) within Immunoglobulin G *β*-sheets molecules. The presence of these two bands, along with the disappearance of bands attributed to citrate ions (at 1590 and 1370 cm^−1^), confirms the successful functionalization of silver nanoparticles with IgG-CF.

Dynamic light scattering analysis was performed to determine the size of silver nanoparticles functionalized with IgG molecules, their numerical and volume share, and the intensity of scattered light. The histogram of dynamic light scattering (DLS) of the AgNPs-IgG bionanosystem reveals a diameter of ~ 30 nm for AgNPs, while AgNP-IgG-CF records an average diameter of ~ 47 nm and a polydispersity index of 0.380 (Fig. 2C).

Atomic force microscopy imaging represents a valuable, direct technique for the evaluation of different conjugation approaches at the level of the individual molecules^17^ In Fig. 2D the AFM image for the AgNP-IgG-CF synthesized bionanostructure is displayed. The main morphological characteristic of the bionanostructure was the spherical shape as seen in Fig. 2D.

### Apoptosis detection

**Figure 3:**
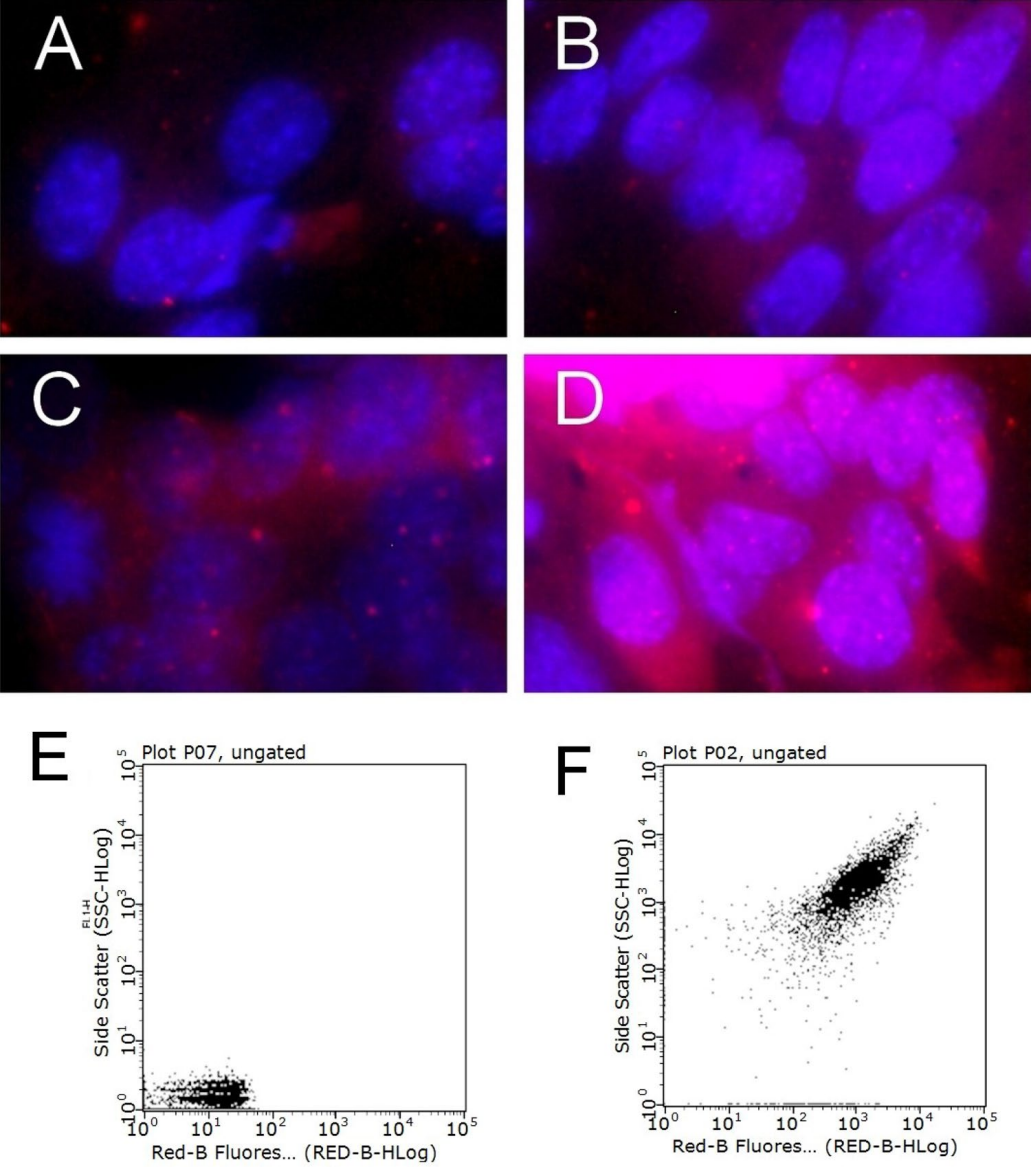
Annexin V staining. A dose-dependent necrosis was obtained for treatment with high concentrations of AgNPs-IgG (control: (**A**) 5 µg/mL: (**B**), 10 µg/mL: (**C**), 50 µg/mL (**D**)). Flowcytometry analysis: control: (**E**) 50 µg/mL: (**F**) *Note* Control sample followed standard cell culture conditions. Test cells were treated with IgG-AgNPs with different concentrations 5, 10, and 50 µg/mL, respectively and laser irradiated. Consequently, all samples were stained with annexin-cy3 for 5 min (RT, dark). Nucleus staining was also performed using DAPI blue-fluorescence. Control sample (no IgG-AgNps exposure, no irradiation). No red fluorescence was observed. Exposure to 5 µg/mL IgG-AgNps (1 h, 37 °C), followed by laser excitation (2 min, 808 nm, 2 W/cm^2^). Red fluorescence is displayed in a reduced number of cells, granular aspect coming from limited number of PS groups exposed on the outer surface of the membrane. Exposure to 10 µg/mL IgG-AgNps (1 h, 37 °C), followed by laser excitation (2 min, 808 nm, 2 W/cm^2^), increased the number of early-apoptosis entrance cells. Red fluorescence aspect is diffuse, suggesting intense PS translocation process. Exposure to 50 µg/mL IgG-AgNps (1 h, 37 °C), followed by laser excitation (2 min, 808 nm, 2 W/cm^2^). The majority of cells present intense, diffuse, red Cy3 fluorescence covering the entire outer surface of the membrane suggesting a highly intense pro-apoptotic effect. Magnification: 60 × .

To assess the targeting of apoptosis pathways following AgNPs-IgG + CF laser treatment, we examined the translocation of phosphatidylserine from the inside to the outside of pancreatic cancer cell membranes using an Annexin V staining kit. Apoptosis induced by silver nanoparticles results in various morphological and biophysical changes within the targeted cells, detectable through various techniques. Given annexin’s high affinity for phosphatidylserine, we could measure the apoptosis rate in targeted cells—an essential consideration in designing cancer therapies. (Fig. 3).

The death of PANC-1 cancer cells due to the activation of apoptotic pathways was significantly higher than in control cells, and this effect was concentration-dependent. Thus, dose-dependent necrosis was observed with high concentrations of AgNPs-IgG (5, 10, 50 µg/mL). The highest intensity of stimulated apoptosis, indicated by red fluorescence (resulting from the coupling of Cy3—annexin V), was observed in samples treated with 50 µg/mL AgNPs-IgG. Flow cytometry analysis revealed a substantial increase in red fluorescence intensity compared to the control group, indicating a robust induction of apoptosis following AgNPs-IgG treatment (at concentrations greater than 10 µg/mL), and laser irradiation demonstrated a significant difference (chi-square, *p* < 0.05).

### Photothermal treatment with AgNPs-IgG-CF causes Golgi apparatus dysfunction with consequent activation of caspase 3

**Figure 4:**
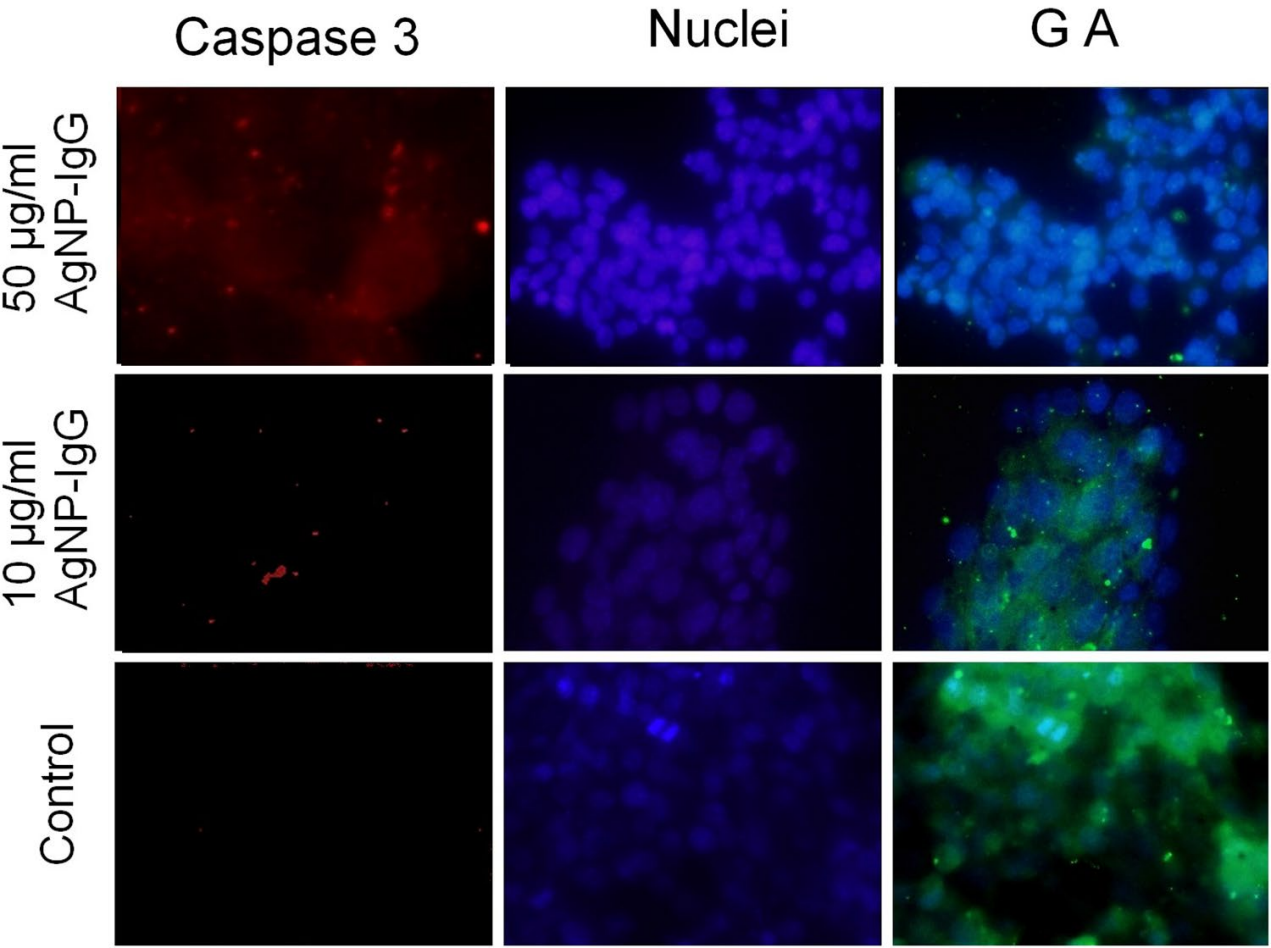
Photothermal treatment with AgNPs-IgG-CF causes Golgi apparatus collapse with subsequent activation of caspase 3. *Note* Middle row: Exposure to 10 µg/mL IgG-AgNPs (1 h, 37 °C), followed by laser excitation (2 min, 808 nm, 2 W/cm^2^). PANC-1 cells: Green fluorescence is less visible, with mild disruption of the Golgi and initiation of caspase 3 apoptosis (red fluorescence). Upper row: Exposure to 50 µg/mL IgG-AgNPs (1 h, 37 °C), followed by laser excitation (2 min, 808 nm, 2 W/cm^2^). PANC-1 cells: Green fluorescence is missing, with complete disruption of the Golgi and full activation of Caspase 3 apoptosis. Lower row: Epithelial cells: Green fluorescence is visible, suggesting that cells function normally. Magnification: 60 × .

We next explored the impact of our treatment on the Golgi apparatus, an organelle commonly targeted in cancer therapy. As depicted in Fig. 3, the Golgi apparatus of PANC-1 cells was locally affected by photothermal ablation combined with AgNPs-IgG-CF. After treatment with 50 µg/mL AgNPs-IgG-CF (1 h, 37 °C), a complete collapse of the Golgi apparatus, a key organelle in cellular function, was observed. This physical damage to the Golgi compartment was also noted when AgNPs-IgG-CF was administered at lower concentrations in a dose-dependent manner (Fig. 4).

The ability of LASER treatment with AgNPs-IgG to cause Golgi dispersal prompted us to further assess the effects of this therapeutic method on the caspase 3 activation pathway in pancreatic cancer cells. Fluorescence microscopy was employed to display the cytoplasmic distribution of caspase 3 in PANC-1 cells with or without AgNPs-IgG-CF treatment. The immunostaining signal of caspase 3 was represented by the presence of green dots in the cytoplasmic region. Activated.

Caspase 3 increased after 5 h of photothermal treatment with 50 µg/mL AGNPs-IgG-CF; furthermore, the intracellular distribution pattern drastically changed, accumulating in the vicinity of the nucleus. In parallel, the Golgi signal in these cells was completely absent, strongly suggesting the complete inactivation of this structure. This finding aligns with other studies describing a specific apoptotic mechanism in malignant cells caused by ER/Golgi stress followed by caspase-3 pathway activation.

### Evaluation of cellular toxicity following AgNPs-IgG-CF administration

Due to safety considerations, before testing the in vitro response of AgNP-IgG-CF -treated cells to laser irradiation, we examined if simple administration of AgNP-IgG-CF induce significant toxicity in PANC-1 cells. Thus, AGNPs-IgG were administered in PANC-1 cells and epithelial cells at different concentrations and different incubation intervals. The degree of apoptosis in cells following AgNP-IgG-CF administration was assessed using flow cytometry combined with annexin staining. After 5 h of incubation, PANC-1 cancer cells treated with 50 µg/mL AgNP-IgG-CF showed a 1.2% decrease in viability compared to 1.8% for AGNPs (*p* < 0.05). Our data suggest that exposure to the developed AgNPs-IgG itself has not induced the significant activation of early apoptosis.

In a similar manner to identify if simple laser irradiation of cancer cells may activate the apoptosis pathway or intra- cellular necrosis, we used our laser to irradiate for 2 min a cell sample without AgNPs treatment. No signs of cellular necrosis or destruction were identified following irradiation.

### Evaluation of cell necrosis after laser treatment and administration of AgNPs-IgG-CF

To avoid potential errors, the subsequent action involved laser treatment of a cell sample without nanoparticles to a 2 min irradiation using an 808 nm and 2 W laser beam. Notably, no indications of cellular mortality were observed following the irradiation.

Next we assessed the impact of our proposed treatment on Panc-1 cells using MTT assay as described above.

**Figure 5:**
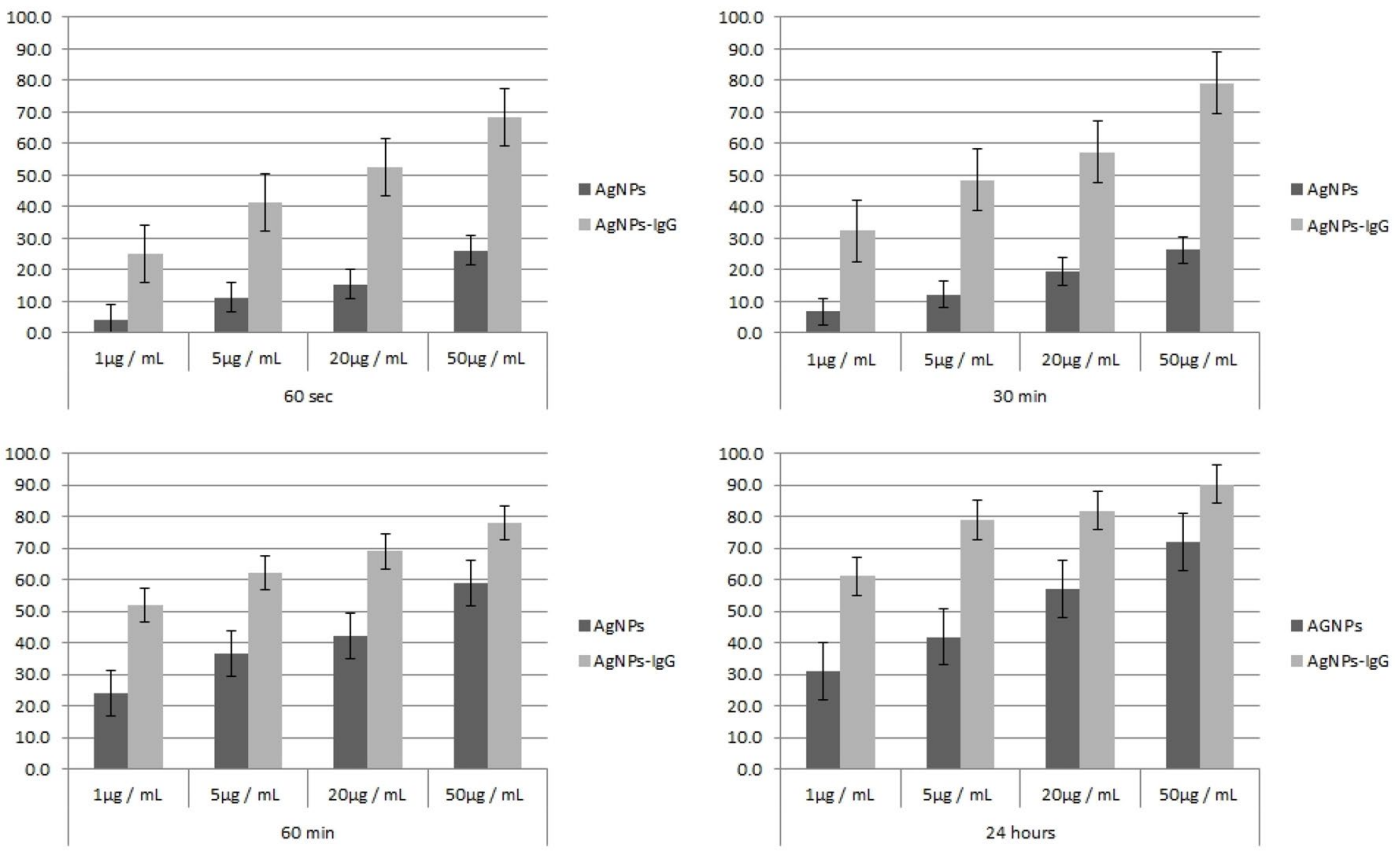
Evaluation of cell viability by MTT assay after treatment at different concentrations and different time intervals. Absorbance values were significantly different from the control group.

As seen in Fig. 5 the MTT analysis of survival rate after laser irradiation of AgNPs-IgG-CF of malignant cells decreased from 68.2% (for 50 µg/mL) to 25.2% (1 µg/mL) at 60 s, while at 30 min the therapeutic rate decreased from 79.1% (50 µg/mL) to 32.3% (1 µg/mL), *p* value < 0.05. In contrast, the lysis rate of epithelial cells treated in similar conditions was significantly lower; with necrotic rates varying from 26.1% (50 µg/mL) to 4.2% (1 µg/mL) for 60 s, 26.3% (50 µg/mL) to 6.8% (1 µg/mL) for 30 min for irradiated econtrol cells. The necrotic rates between the two cell lines following the LASER AgNPs-IgG-CF treatment were also significantly different (*p* values were < 0.05 in all groups) after incubation for 60 min, at low/medium concentrations of AgNPs-IgG (78.1%—50 µg/mL, for PANC-1 cells, 59.1%—50 µg/mL, for control cells).

## Discussions

Nanoparticles have the capability to be specifically engineered to gather in tumors due to their distinct attributes, including their minute size and surface qualities. Upon reaching the tumor site, a laser may be utilized to elevate their temperature, thereby inducing intracellular necrosis. Plasmonic activity is observed in silver nanoparticles, denoting a collective movement of liberated electrons within a metal when exposed to light. The exceptional optical features of silver nanoparticles stem from surface plasmon resonance (SPR), which emerges when the incident light frequency aligns with the inherent frequency of the conduction electrons on the nanoparticles’ surface. Laser-induced hyperthermia therapy represents a promising strategy for treating cancer as it can effectively pinpoint cancerous cells while mitigating harm to healthy tissues. Nevertheless, further investigation is imperative to refine this method and guarantee its safety and efficacy in cancer therapy. Immunoglobulin G, an antibody molecule synthesized by the immune system, serves to defend against bacterial and viral invasions. The prospect of leveraging Immunoglobulin G molecules as active agents that target Immunoglobulin G receptors on malignant cell surfaces has been extensively examined in the field of Oncology. It has been a successful strategy in cancer immunotherapy, with several monoclonal antibody-based therapies approved for various types of cancer.

The laser ablation with nanoparticles approach has several potential advantages over traditional laser ablation therapy, including increased selectivity and reduced damage to surrounding healthy tissue. Additionally, the use of nanoparticles can enhance the delivery and distribution of heat within the tumor tissue, potentially improving the overall effectiveness of the treatment.

Several studies have shown promising results for the use of laser ablation with nanoparticles in preclinical models of pancreatic cancer^6^. However, more research is needed to optimize the design of nanoparticles and laser parameters and to evaluate the safety and efficacy of this approach in clinical trials.

The primary aim of this research was to formulate and evaluate a novel treatment approach for pancreatic carcinoma in humans. Previous literature provides initial evidence supporting the impact of Immunoglobulin G on the progression of tumors. It is widely recognized that Immunoglobulin G plays a crucial role as a key component in the angiogenic process of cancerous growths, facilitating tumor proliferation by inducing angiogenesis via a fivefold rise in the production of morpholine-modified oligonucleotides MO VEGF (Morpholino-modified antisense oligonucleotides)^18^. On the other hand side silver nanoparticles are one of the most biocompatible nanoparticles and at the current time several treatment based on AgNPs are approved for human treatment especially as antimicrobial agents^19^.

In this context, in an additional experiment, PANC-1 cells and epithelial cells were treated with different concentrations of AgNPs-IgG for varying periods to interpret cytotoxicity. Consistent with data published by other authors, we have shown that even at high concentrations, compounds based on silver nanoparticles do not exhibit significant cytotoxic effects.

However, toxicity, an important health issue, could be overcome by the administration of small amounts of nanocom- posites or by bioconjugation with biological moieties^20^. Next, we explored the role of AgNPs-IgG-CF as plasmonic-heat- inducing agents under laser exposure. This method relies on the internalization of AgNPs-IgG-CF inside the cells and their special optical absorption capacities, especially under irradiation in the near-infrared range, where biological systems have low absorption and high transparency. The optoelectronic transition on the external surface of the nanoparticle generates thermal energy, which diffuses rapidly into the subcellular compartments, causing cellular lysis at the molecular level.

By subjecting PANC-1 cells to continuous laser exposure, statistically significant distinctions were observed between AgNPs-IgG-CF and AGNPs in terms of the degree of cell lysis post-irradiation (*p* < 0.05) at low concentrations during both 60 s and 30 min of incubation. This discovery holds particular significance for low concentrations of AgNPs-IgG, such as those found in plasma following in vivo administration. To the best of our knowledge, this represents the initial evidence of laser-induced thermal ablation of pancreatic cancer cells utilizing IgG-AGNPs. In biological systems, the processes of molecular membrane transport are of very short duration, in terms of seconds. Therefore, our data could have a significant clinical impact when using AgNPs-IgG-CF for the photothermal therapy of pancreatic cancer cells. We have shown here that caspase 3 function as a triggering caspase and this process is initiated in the Golgi apparatus. Following treatment with AgNPs-IgG-CF + laser, caspase 3 relocated into the cytoplasm, an observation with high clinical importance since it is known that translocation of caspase 3 sites by cleavage of the structural protein golgina-160 alters the mechanism of chemoresistance. In the shed light of new anticancer therapeutics discoveries the pathways involving the Golgi apparatus targeting and cell apoptosis are recognized as potential drug targets^21^. The presented results could be a step forward toward the development of new thermal nanotherapies against pancreatic cancer using nanolocalized thermal ablation by laser activation. However, studies are needed to carefully evaluate unexpected toxic reactions and biological interactions of AgNPs-IgG-CF when administered to living organisms (Supplementary Fig. 1).

## Conclusions

Pancreatic cancer is a highly aggressive and often difficult-to-treat cancer. Nanoparticles have emerged as a promising tool for pancreatic cancer treatment due to their ability to selectively target cancer cells and enhance drug delivery to tumors. Despite the promise of IgG-nanoparticle-based therapies for pancreatic cancer, more research is needed to optimize their design and delivery to improve their efficacy and safety in clinical settings.

### Supplementary Information

Supplementary Figure 1.

Supplementary Information.

## Data Availability

The datasets generated during and/or analysed during the current study are available from the corresponding author on reasonable request.
