# Supplementary material for: In vitro photothermal therapy of pancreatic cancer mediated by immunoglobulin G-functionalized silver nanoparticles
Source: Sci Rep. 2024 Jun 22;14:14417. doi: 10.1038/s41598-024-63142-4 (PMC11193743; doi:10.1038/s41598-024-63142-4)
Supplement: Supplementary file 1 — Supplementary Figure 1. [file 41598_2024_63142_MOESM1_ESM.docx]

| 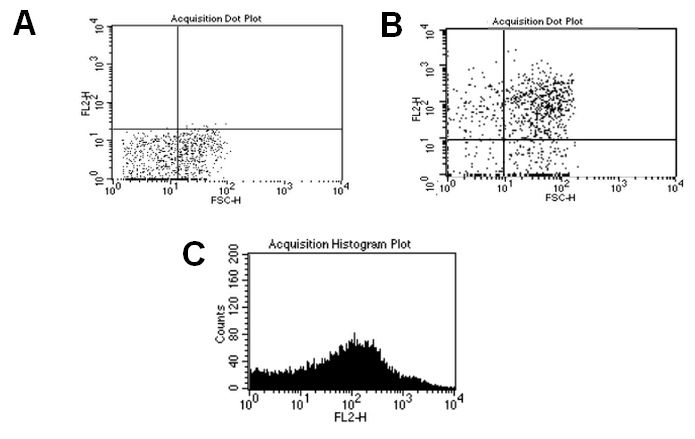 |
| --- |
| Figure 1 supplementary. Upper row scatter plots of FSC-Height (cell size) versus FL2-Height (red fluorescence-PI content). A: Control B: Exposure to 50µg/mL IgG-AgNps (1 hour, 37◦C), followed by laser excitation (2 minutes, 808 nm, 2W/cm2) C: Flow cytometry histogram of the PI fluorescent intensities produced by malign cells following exposure to 50µg/mL IgG-AgNps (1 hour, 37◦C), followed by laser excitation (2 minutes, 808 nm, 2W/cm2). |
