# Supplementary material for: In vitro photothermal therapy of pancreatic cancer mediated by immunoglobulin G-functionalized silver nanoparticles
Source: Sci Rep. 2024 Jun 22;14:14417. doi: 10.1038/s41598-024-63142-4 (PMC11193743; doi:10.1038/s41598-024-63142-4)
Supplement: Supplementary file 2 — Supplementary Information. [file 41598_2024_63142_MOESM2_ESM.docx]

**Assessment of necrosis following photothermal treatment using propidium Iodide staining**

Propidium iodide (PI) , a membrane-impermeable dye, can enter damaged cell membranes and emit red fluorescence upon binding to DNA. Its widespread use in assessing cell damage is well-documented. Evaluating cell viability is crucial for monitoring responses to cytotoxic agents or environmental stressors. Cell death processes can be classified as apoptotic or necrotic, with necrosis characterized by loss of membrane integrity and ion transport capabilities. Necrosis typically affects entire regions of cells, triggering an inflammatory response in adjacent healthy tissues due to the release of cellular debris. Conversely, apoptosis involves intact cell and mitochondrial membranes initially, followed by cytoplasmic condensation and nuclear fragmentation into DNA segments.

Propidium iodide staining was also conducted for the quantification of necrotic cells. In order to assess the photothermal therapy (PTT) efficiency in vitro, cells were exposed to laser irradiation as described in the methods section, washed with PBS, stained with PI at 37°C for 20 minutes in the absence of light. Subsequently, the harvested cells were aliquoted up to 1 x 10^6^ cells/100 μL into FACS tubes, washed with 2 mL PBS (or HBSS), centrifuged at 300 x g for 5 minutes, and the supernatant was removed. The cells were then resuspended in 100 μL of Flow Cytometry Staining Buffer. To optimize flow cytometer parameters for PI, 10 μL of PI staining solution was added to unstained cells in a control tube and incubated for 1 minute in darkness. The PI fluorescence was analyzed using the FL-2 channel of a BD FACScan flow cytometer.

Flow cytometry is a valuable tool for quantifying cell death in cell suspensions. During early apoptosis, phosphatidylserine (PS) is exposed on the outer cell membrane, detectable by PS-binding proteins like Annexin V. Necrotic cells allow PI penetration into nuclear DNA, emitting red fluorescence. Representative FACS dot plots gated on PANC-1 cells illustrate the lack of red fluorescence (FL2) in viable control cells (Figure 1A, top right) and increased red fluorescence indicative of nuclear disruption and necrosis post photothermal treatment (Figure 1B, C). Cells exposed to 50µg/mL IgG-AgNps (1 hour, 37°C) followed by laser excitation (2 minutes, 808 nm, 2W/cm2) exhibited signs of cellular necrosis, confirming the efficacy of IgG-AgNps laser treatment on pancreatic cancer cells in vitro.
